# Pseudomonas mendocina Urinary Tract Infection: A Case Report and Literature Review

**DOI:** 10.7759/cureus.23583

**Published:** 2022-03-28

**Authors:** Thy Vo, Nodari Maisuradze, David Maglakelidze, Tanisha Kalra, Isabel M McFarlane

**Affiliations:** 1 Internal Medicine, State University of New York (SUNY) Downstate College of Medicine, Brooklyn, USA; 2 Internal Medicine, State University of New York (SUNY) Downstate Medical Center, Brooklyn, USA; 3 Internal Medicine, Coney Island Hospital, Brooklyn, USA; 4 Internal Medicine, State University of New York (SUNY) Downstate Medical Center, New York, USA

**Keywords:** urinary tract infection, pseudomonas infections, pseudomonas mendocina, mendocina, pseudomonas

## Abstract

*Pseudomonas mendocina *is a Gram-negative bacillus from the family Pseudomonadaceae. The first* P. mendocina*-related infection was reported in 1992. Although a rare cause of infections, *P. mendocina *has been known to cause severe infections that require intensive treatment. We present the first documented case of urinary tract infection caused by *P. mendocina. *

An 83-year-old male with a past medical history of diabetes, hypertension, coronary artery disease, and prostate cancer with bone metastases, currently being treated with abiraterone and prednisone, presented with subjective fever, fatigue, altered mental status, dysuria, and hematuria of one-week duration. He was found to have a complicated urinary tract infection with an incidental asymptomatic COVID-19 infection on admission. The patient was empirically treated with ceftriaxone and switched to cefepime for broader coverage on day two of hospitalization. Urine culture reported the presence of *P. mendocina *with resistance only to fluoroquinolones. Ceftriaxone was reinstated. The patient was successfully treated with a seven-day course of ceftriaxone (days 1-3, days 6-7) and cefepime (days 4-5) but continued to remain inpatient for a later symptomatic COVID-19 pneumonia with discharge on day 15.

The majority of *P*. *mendocina *infections present as skin and soft tissue infections, infective endocarditis, meningitis, and bacteremia. Ours is the first documented case of urinary tract infection caused by *P. mendocina, *particularly in an immunocompromised COVID-19 patient, and the second to report *P. mendocina* with resistance to fluoroquinolones. This report contributes to the growing literature regarding *P. mendocina*-related infections.

## Introduction

*Pseudomonas mendocina* is a motile, Gram-negative, aerobic bacillus, belonging to the family Pseudomonadaceae [1]. *P. mendocina* is found ubiquitously in soil and water and can grow in various temperatures ranging from 25 ºC to 42 ºC [1,2]. *P. mendocina* is a rare cause of human infections. The first case of *P. mendocina*-related infection was reported in Mendoza, Argentina in 1992 [2]. Since then, *P. mendocina*-related infections have been seldomly documented in Asia [3-7], Europe [8-10], Middle East [11,12] North America [13-16], and South America [17]. Despite its low incidence of pathogenicity, *P. mendocina* has been known to cause severe infections, requiring hospitalization and intensive treatment.

A systematic review of current literature reveals endocarditis, meningitis, and bacteremia to be the most common presentations of *P. mendocina* infections [18]. In the United States, there have been four documented cases of *P. mendocina-*related infections, which include three reports of bacteremia and one report of infective endocarditis [13-16]. In this case report, we present the first documented case of urinary tract infection caused by *P. mendocina*.

## Case presentation

An 83-year-old male presented to the emergency department with subjective fever, fatigue, altered mental status, dysuria, and hematuria of one-week duration. His past medical history included diabetes, hypertension, coronary artery disease, and prostate cancer with bone metastases. He had been receiving prostate cancer treatment for at least 10 years, consisting of prednisone 5 mg daily and abiraterone 1000 mg daily with an injection every three months.

On arrival, he had a temperature of 38.7 °C (101.7 °F), a heart rate of 79/minute, a blood pressure of 119/72 mmHg, and a respiratory rate of 18/minute with an oxygen saturation of 99% on room air. On the physical exam, he appeared cachectic with bilateral pale conjunctivae. Oropharyngeal mucous membranes were dry. Suprapubic or costovertebral tenderness was not elicited on the abdominal exam. A genitourinary exam revealed no masses, blood, or discharge at the penile meatus. There was no edema, erythema, lesions, or warmth noted in the penis or scrotum.

**Figure 1:**
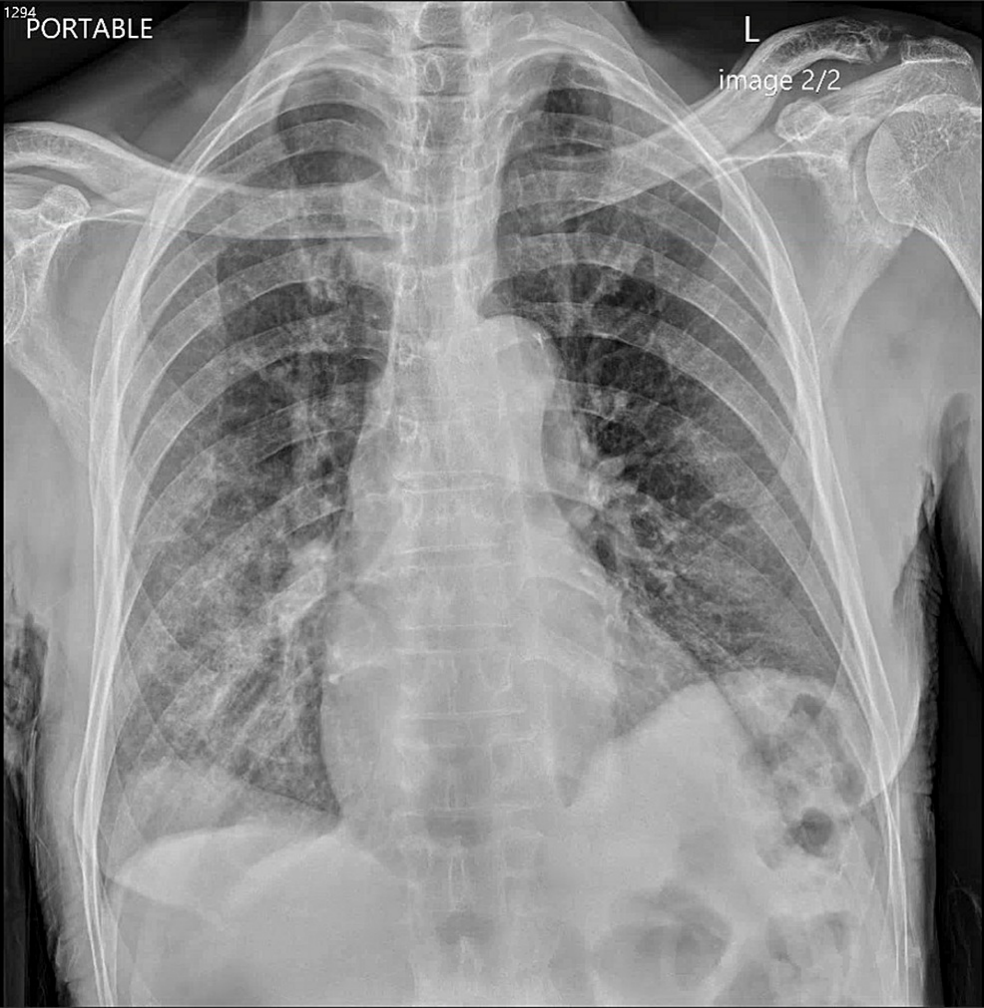
Chest X-ray of our patient on admission

Laboratory evaluation revealed pancytopenia with a WBC count of 2530/μL, absolute neutrophil count of 1200/μL, hemoglobin of 11.1 g/dL, and a platelet count of 106/μL (Table 1). A urinalysis demonstrated RBC 16/hpf, WBC 13/hpf, protein 100 mg/dL, urobilinogen 4 mg/dL, with small amounts of blood, negative nitrite, trace leukocyte esterase, and few bacteria (Table 2). A standard pre-admission COVID-19 screening test was positive. Chest X-rays showed mild right basilar hazy reticulation and minimal left basilar opacification (Figure 1). The patient was admitted with a complicated urinary tract infection and a concurrent asymptomatic incidental COVID-19 infection. Therapy was initiated with ceftriaxone.

**Table 1:** Laboratory findings of our patient WBC: white blood cell, RBC: red blood cell, MCV: mean corpuscular volume, MCH: mean cell hemoglobin, MCHC: mean cell hemoglobin concentration, RDW: red cell distribution width

Laboratory test	On admission	Day 2 of admission	Day 7 of admission	Reference ranges
WBC (×10^3^ μL)	2.53	5.06	4.68	3.50–10.80
RBC (×10^6^ μL)	3.59	3.54	3.43	4.70–6.10
Hemoglobin (g/dL)	11.1	10.7	10.8	14.0–18.0
Hematocrit (%)	32.2	31.7	30.8	42.0–52.0
MCV (fL)	89.9	89.5	89.7	80.0–95.0
MCH (pg)	31.0	30.3	31.6	27.0–31.0
MCHC (%)	34.5	33.9	35.2	33.0–37.0
RDW	14.8	14.8	14.6	11.5–14.5
Platelets (×10^3^ μL)	106	126	198	130–400
Neutrophil (%)	47.3	84.9	80.5	40.0–74.0
Lymphocyte (%)	42.2	9.9	14.6	19.0–48.0
Monocyte (%)	6.4	4.6	3.3	0.0–9.0
Eosinophil (%)	1.3	0.0	0.4	0.0–7.0
Basophil (%)	0.6	0.1	0.1	0.0–1.5
Neutrophil (×10^3^ μL)	1.2	4.3	3.8	1.7–7.0
Lymphocyte (×10^3^ μL)	1.1	0.5	0.7	0.9–2.9
Monocyte (×10^3^ μL)	0.2	0.2	0.2	0.0–1.0
Eosinophil (×10^3^ μL)	0.03	0.0	0.0	0.0–0.80
Basophil (×10^3^ μL)	0.0	0.0	0.0	0.0–0.2

**Table 2:** Urinalysis of our patient

Component	On admission	Day 7 of admission	Reference ranges
Dipstick analysis			
Appearance	Cloudy	Cloudy	Clear
Color	Amber	Yellow	Yellow
Glucose level	Negative	Negative	Negative
Bilirubin (mg/dL)	Negative	Negative	Negative
Ketones	5	5	Negative
Specific gravity	1.017	1.017	1.005–1.030
Blood	Small	Moderate	Negative
pH	6.0	5.0	5.0–7.0
Protein UA (mg/dL)	100	100	Negative
Urobilinogen (mg/dL)	4.0	<2.0	<2.0
Nitrite	Negative	Negative	Negative
Leukocyte esterase	Trace	Negative	Negative
Urine microscopy			
RBC (per high-power field)	16	2	0–4
WBC (per high-power field)	13	4	0–5
Bacteria (per high-power field)	Few	None	None
Squamous epithelial cells (per high-power field)	Rare	Rare	None

The patient remained febrile during the first three days of admission with a maximum temperature of 38.5 °C (101.4 °F). Absolute neutrophil count and WBC increased to 4300/μL and 5060/μL, respectively, on day 2 of admission (Table 1). Preliminary urine culture reported >100,000 CFU/mL of Gram-negative rods. Multiple blood cultures were negative for the presence of bacteria. Due to the persistence of fever, the antibiotic coverage was broadened to cefepime while awaiting final speciation.

A urine culture report from our hospital microbiology department demonstrated the presence of *P. mendocina* with resistance to ciprofloxacin and levofloxacin. The provided antibiotic susceptibilities of the *P. mendocina* isolate from our patient are summarized in Table 3. MICs were not available to us retrospectively. Following the report, cefepime was discontinued and ceftriaxone was reinstated. By day 7 of admission, the patient no longer endorsed hematuria or dysuria. Absolute neutrophil count and WBC were 3800/μL and 4680/μL on day seven of admission (Table 1). The patient received a total of seven days of ceftriaxone (days 1-3, days 6-7) and cefepime (days 4-5). The patient was intermittently febrile, likely due to underlying malignancy and COVID-19 infection. A repeat urinalysis obtained due to the persistence of fevers showed no bacteria, with negative nitrite, negative leukocyte esterase, and moderate blood (Table 2). Complete resolution of urinary symptoms post-antibiotic therapy suggested that these symptoms were due to his urinary tract infection and less likely his prostate cancer. The hospital course was complicated by hypoxic respiratory failure due to COVID-19 infection, requiring prolonged hospitalization. The patient was successfully discharged after a 15-day hospital stay.

**Table 3:** Antibiotics susceptibility profile of isolated Pseudomonas mendocina

Antibiotics	Susceptibility
Amikacin	Susceptible
Aztreonam	Susceptible
Cefepime	Susceptible
Ceftriaxone	Susceptible
Ciprofloxacin	Not susceptible
Gentamicin	Susceptible
Levofloxacin	Not susceptible
Meropenem	Susceptible
Piperacillin/tazobactam	Susceptible
Tetracycline	Susceptible
Tobramycin	Susceptible
Trimethoprim/sulfamethoxazole	Susceptible

## Discussion

*Pseudomonas aeruginosa* has been known to cause severe nosocomial and opportunistic infections in immunocompetent and immunocompromised adults [19]. *P. mendocina*, however, is a rare cause of human infections and is less frequently reported in the literature.

A literature search was performed on PubMed using the terms "Pseudomonas mendocina" and "Pseudomonas mendocina infection." The query returned 14 case reports of *P. mendocina*-related infections in humans. Additional case reports were identified by cross-referencing previously discovered case reports. A total of 20 cases of *P. mendocina*-related infections were documented. Seven were from Asia (Taiwan [3,4], Singapore [5,6], and India [7]), three were from Europe (Denmark [8], France [9], and Portugal [10]), two were from the Middle East (Israel [11] and Turkey [12]), four were from North America (USA [13-16]), and two were from South America (Argentina [2,17]. *P. mendocina* can cause various infections, including infective endocarditis, meningitis, skin and soft tissue infections (burn wound infections, leg wound infections, and spondylodiscitis), peritonitis, septic arthritis, osteomyelitis, and bacteremia. Ours is the fifth case report of *P. mendocina* infection in the United States and the first documented case of *P. mendocina* urinary tract infection. A systematic literature review by Ioannou and Vougiouklakis in 2020 demonstrated that previous cases of *P. mendocina* had low mortality [18]. No deaths directly attributed to *P. mendocina* were reported.

**Table 4:** Current literature reports on P. mendocina

Publication year	Author	Location	Age	Sex	Comorbidities	Infection type	Antibiotic resistance
1992	Aragone et al.[2]	Argentina	63	Male	Diabetes mellitus type 2, aortic valve replacement, poliomyelitis	Infective endocarditis	Ampicillin, cephalothin
2001	Johansen et al. [8]	Denmark	28	Female	Situs inversus, double-outlet right ventricle, ventricular septal defect (VSD), pulmonary stenosis, multiple cardiovascular surgeries	Infective endocarditis	No available data, culture unable to be obtained from abscess
2005	Chi et al. [4]	Taiwan	65	Male	Alcoholic hepatitis, chronic renal disease	Spondylodiscitis	Trimethoprim/sulfamethoxazole
2007	Mert et al. [12]	Turkey	36	Male	Mental retardation	Infective endocarditis	No known resistance
2011	Suel et al. [9]	France	79	Female	Atrial fibrillation, transient ischemic attack, hypertension	Infective endocarditis	No known resistance
2011	Nseir et al. [11]	Israel	31	Male	Healthy	Bacteremia	Ceftriaxone and aztreonam
2013	Howe et al. [6]	Singapore	86	Female	Vertebral compression fractures, tibial plateau stress fracture	Osteomyelitis	No available data, polymicrobial infection
2013	Chiu and Wang [5]	Singapore	34	Male	Healthy	Septic arthritis	Ampicillin ampicillin/sulbactam
2016	Rapsinski et al. [15]	United States	57	Male	Gout, chronic alcohol use	Infective endocarditis	Ampicillin/sulbactam, cefazolin
2017	Jerónimo et al. [10]	Portugal	22	Male	Chronic kidney disease, peritoneal dialysis	Peritonitis	No available data
2018	Almuzara et al. [17]	Argentina	56	Male	Alcohol use disorder, vascular insufficiency	Burn wound infection	No known resistance
2018	Almuzara et al. [17]	Argentina	36	Male	Alcohol use disorder	Burn wound infection	No known resistance
2018	Huang et al. [3]	Taiwan	55	Male	Diabetes mellitus type 2, buccal cancer, community-acquired infection	Meningitis	No known resistance
2018	Huang et al. [3]	Taiwan	66	Female	Spontaneous intracerebral hemorrhage, external ventricular drainage	Meningitis	No known resistance
2018	Huang et al. [3]	Taiwan	79	Male	Chronic obstructive pulmonary disease, respiratory failure, nosocomial infection	Meningitis	No known resistance
2018	Huang et al. [3]	Taiwan	78	Female	Healthy	Meningitis	No known resistance
2019	Gani et al. [16]	United States	63	Male	Resistant HIV/AIDS	Bacteremia	No resistance against cefepime, ceftazidime, levofloxacin, meropenem; resistance against piperacillin/tazobactam unable to be determined
2020	Goldberg et al. [14]	United States	72	Male	End-stage renal disease, immunoglobulin A (IgA) nephropathy, atrial fibrillation, heart failure with reduced ejection fraction, obesity, chronic venous stasis	Bacteremia	No known resistance
2021	Ezeokoli et al. [13]	United States	81	Male	Coronary artery disease, atrial fibrillation, heart failure, chronic kidney disease, diabetes mellitus type 2, CVA	Bacteremia	No known resistance
2021	Gupta et al. [7]	India	53	Male	Diabetes mellitus type 2, asthma	Leg wound infection	Ciprofloxacin, ceftazidime, amikacin, piperacillin-tazobactam, aztreonam
2022	This case report	United States	83	Male	Diabetes mellitus type 2, hypertension, coronary artery disease, prostate cancer, COVID-19 pneumonia	Urinary tract infection	Ciprofloxacin, levofloxacin

Previous cases of *P. mendocina* infections reported successful treatments with various antibiotics, including penicillins, aminoglycosides, carbapenems, cephalosporins, fluoroquinolones, and trimethoprim-sulfamethoxazole [1-17]. Across all cases, third- or fourth-generation cephalosporins and fluoroquinolones were commonly used agents for the treatment of *P. mendocina*. Documented *P. mendocina* isolates have shown susceptibility to non-traditional antipseudomonal antibiotics, such as ampicillin, cefazolin, and trimethoprim-sulfamethoxazole, allowing for a broader range of antibiotic selection compared to that of *P. aeruginosa*. Some cases reported susceptibility to all antibiotics tested, including aminoglycosides, ampicillin, carbapenems, later-generation cephalosporins, fluoroquinolones, and piperacillin/tazobactam [3,9,12-14,17]. However, some cases are also reported to have resistance to a variety of antibiotics, including ampicillin, amikacin, aztreonam, cephalothin, cefazolin, ceftazidime, aztreonam, ciprofloxacin, piperacillin-tazobactam, and trimethoprim-sulfamethoxazole [2,4-6,7,11,15]. Gupta et al. reported an isolate that had resistance to multiple antibiotics, including ciprofloxacin, ceftazidime, amikacin, piperacillin-tazobactam, and aztreonam [7]. Our isolate was resistant to ciprofloxacin and levofloxacin, making this the second documented isolate of *P. mendocina* that showed resistance to fluoroquinolones. Our patient was successfully treated with a seven-day course of ceftriaxone and cefepime.

*P. mendocina* can infect both immunocompromised and immunocompetent hosts. Most of the reported *P. mendocina*-related infections occurred in immunocompetent adults with several comorbidities, as shown in Table 4. A few cases were reported in immunocompromised adults. Gani et al. reported bacteremia in a patient with resistant HIV/AIDS [16]. Huang et al. reported meningitis in a patient with a history of diabetes mellitus type 2 and buccal cancer [3]. Gupta et al. previously reported a wound infection in a patient with diabetes mellitus type 2 and a prolonged history of asthma and intermittent corticosteroid use [7]. Our patient had a 10-year history of prostate cancer with bone metastases and was receiving treatment with abiraterone 1000 mg daily with an injection every three months and prednisone 5 mg daily. His immune system might be compromised due to his prostate cancer and treatment. It can be argued that *P. mendocina* caused an opportunistic urinary tract infection in our immunocompromised patient.

Recent literature has documented the increase in opportunistic infections in patients with concomitant COVID-19 infections [20]. In particular, fungal infections remain the most common opportunistic infections amongst immunocompromised adults with COVID-19 [21,22]. Studies have also reported the association of COVID-19 with bacterial infections [23]. These infections are classified as nosocomial infections and are associated with increased morbidity and mortality among COVID-19 patients. In these patients, *Staphylococcus aureus* and *Haemophilus influenzae* are the most common bacterial infections [24]. *Mycoplasma pneumoniae*, *Pseudomonas aeruginosa*, and *Legionella pneumophila* are other important bacterial pathogens that were detected among COVID-19 patients [22]. However, to date, no report has shown infection of *P. mendocina* in those with COVID-19 infections. We suspect that the combination of active COVID-19 infection and our patient's cancer status and treatment increases the risk for *P. mendocina* infection. 

Multiple sources of *P. mendocina* were proposed but never confirmed in previous case reports. In the first-ever report of *P. mendocina*, Aragone et al. proposed that *P. mendocina* caused infective endocarditis by entering through thorn pricks and handling of damp earth as the patient was a florist with previous aortic valve placement and a permanent pacemaker [2]. Johansen et al. suspected that bacteria was introduced during one of the three cardiac operations, resulting in infective endocarditis [8]. Gupta et al. proposed that *P. mendocina* was present in soil and water, which gained entry into a leg wound when the patient fell while working on his farm [7]. In the case reported by Nseir et al., since the patient owned "a new pet cockatiel that he fed and watered directly from his mouth," the shared drinking water might be the source of *P. mendocina* [11]. In our patient, the source of *P. mendocina* was not identified.

Our case report adds to the current literature regarding *P. mendocina* infections. Although further research is required to identify the underlying pathogenicity and mechanism of *P. mendocina* infections, our report contributes to the growing body of publications that can help guide clinical management and treatment of *P. mendocina* infections in the future.

## Conclusions

*P. mendocina* is a gram-negative bacillus that rarely causes infections in humans. When it does, *P. mendocina* has been known to cause skin and soft tissue infections, infective endocarditis, meningitis, and bacteremia. Our case is the first to report a urinary tract infection caused by *P. mendocina* and the second with fluoroquinolone resistance. In particular, this is also the first report on *P. mendocina* infection in an immunocompromised patient with COVID-19. Our report is the fifth documented case of *P. mendocina*-related infections in the United States, contributing to the growing literature regarding *P. mendocina*-related infections.
